# rs140926439 variant in the Fibronectin FN1 gene protects against Alzheimer’s disease in APOEε4 carriers in the UK Biobank cohort

**DOI:** 10.21203/rs.3.rs-4287946/v1

**Published:** 2024-04-19

**Authors:** Steven Lehrer, Peter Rheinstein

**Affiliations:** Icahn School of Medicine Mount Sinai; Severn Health Solutions

**Keywords:** fibronectin, Alzheimer’s Disease, APOEε4

## Abstract

**Background:**

A protective genetic variant in the fibronectin FN1 gene reduces the odds of developing AD by up to 70%. This variant, rs140926439, seems to prevent the buildup of excess fibronectin at the blood brain barrier. Increased fibronectin levels are typically observed in people with Alzheimer’s Disease (AD), but the protective variant appears to counteract its effects.

**Methods:**

In the current study, we analyzed the relationship of FN1 SNP rs140926439, APOEε4, and AD in the UK Biobank cohort.

**Results:**

When rs140926439 was absent, 0.10% of APOEε2/3 carriers had AD while 0.40% of APOEε4 carriers or homozygotes had AD. This difference was significant (p < 0.001, 2 tail Fisher exact test). When rs140926439 was present, 0.10% of APOEε2/3 carriers had AD while 0.10% of APOEε4 carriers or homozygotes had AD. This difference was insignificant (p = 1). To examine the overall relationship of rs140926439 and APOE isoform to AD, we used the univariate general linear model, AD (present or absent) dependent variable, rs140926439 (present or absent) and APOE isoform (APOEε2/3 or APOEε4 carrier or homozygote) as fixed factors. The effect of rs140926439 was significant (p = 0.030). The effect of APOE isoform was significant (p = 0.034). There was also a significant interaction between rs140926439 and APOE isoform (p = 0.030).

**Conclusion:**

Fibronectin is an adhesive molecule that is essential to wound healing, especially to the production of extracellular matrix and reepithelialization. Some cases of AD may be due to the initiation of the brain wound healing process, often in the absence of any actual wound. NSAIDS may reduce risk of AD because they potently inhibit wound healing. FN1 appears to be a key player in AD, and its protective variant could offer insights into potential therapeutic targets. However, further research is needed to fully understand the intricate mechanisms underlying AD and to develop effective treatments.

## Introduction

Fibronectin is an extracellular matrix (ECM) protein that plays a crucial role in cell adhesion, tissue repair, inflammation, and wound healing. Bhattarai et al have reported a protective genetic variant in the fibronectin FN1 gene that reduces the odds of developing AD by up to 70% [1].

This variant, rs140926439, seems to prevent the buildup of excess fibronectin at the blood brain barrier. Increased fibronectin levels are typically observed in people with Alzheimer’s Disease (AD), but the protective variant appears to counteract its effects [1].

Brain expression of FN1 in cognitively unaffected homozygous APOEε4 carriers is lower than in those with AD, suggesting that FN1 may be involved in AD-related pathology and cognitive decline. Zebrafish models with loss of function mutations in the FN1b gene (the ortholog for human FN1) demonstrated enhanced amyloid clearance, further supporting the role of fibronectin in AD [1].

In the current study, we analyzed the relationship of FN1, APOEε4, and AD in the UK Biobank cohort.

## Methods

The UK Biobank (UKBB) is a large prospective observational study comprising approximately 500,000 men and women (N = 229,134 men, N = 273,402 women), more than 90% white, aged 40–69 years at enrollment. Participants were recruited from across 22 centers located throughout England, Wales, and Scotland between 2006 and 2010 and continue to be longitudinally followed for capture of subsequent health events [2]. This methodology is like that of the Framingham Heart Study [3], with the exception that the UKBB program collects postmortem samples, which Framingham did not. Our UKBB application was approved as UKB project 57245 (S.L., P.H.R.).

UK Biobank: has approval from the Northwest Multi-center Research Ethics Committee (MREC) to obtain and disseminate data and samples from the participants, and these ethical regulations cover the work in this study. Written informed consent was obtained from all participants. Details can be found at www.ukbiobank.ac.uk/ethics.

We analyzed the FN1 SNP rs140926439, position chr2:215424292, a single nucleotide missense variant, C > T, minor allele frequency 0.005, cohort allele count 4793. Single nucleotide polymorphism (SNP) data for rs429358 and rs7412 were used to determine APOE isoforms [4]. Phenome-wide association study (PHEWAS) of rs140926439 was done on PheWeb (https://pheweb.org).

Data processing was performed on Minerva, a Linux mainframe with Centos 7.6, at the Icahn School of Medicine at Mount Sinai. We used PLINK, a whole-genome association analysis toolset, to analyze the UKB chromosome files [5]. Statistical analysis was done with SPSS v 26, (IBM, New York).

## Results

Mean age of 413,127 subjects was 56 ± 8 (mean ± SD), 54% women, 46% men, 95% white British, 15 ± 5 years of education.

Table 1 illustrates the relationship of rs140926439 SNP and APOE isoform to AD. When rs140926439 was absent, 0.10% of APOEε2/3 carriers had AD while 0.40% of APOEε4 carriers or homozygotes had AD. This difference was significant (p < 0.001, 2 tail Fisher exact test). When rs140926439 was present, 0.10% of APOEε2/3 carriers had AD while 0.10% of APOEε4 carriers or homozygotes had AD. This difference was insignificant (p = 1).

To examine the overall relationship of rs140926439 and APOE isoform to AD, we used the univariate general linear model, AD (present or absent) dependent variable, rs140926439 (present or absent) and APOE isoform (APOEε2/3 or APOEε4 carrier or homozygote) as fixed factors. The effect of rs140926439 was significant (p = 0.030). The effect of APOE isoform was significant (p = 0.034). There was also a significant interaction between rs140926439 and APOE isoform (p = 0.030).

**Figure 1:**
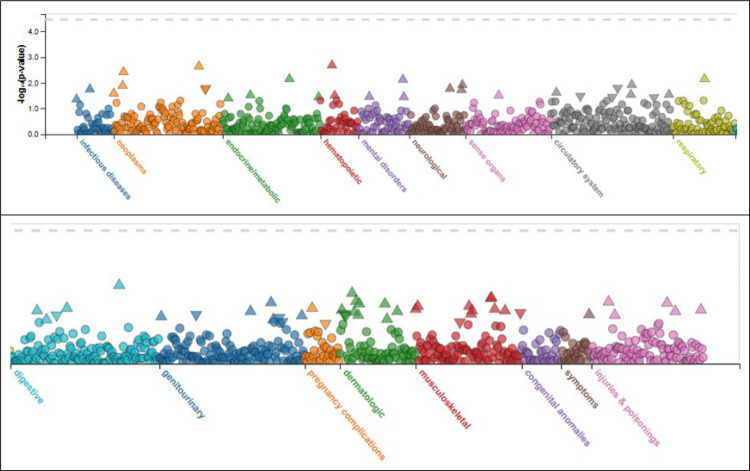
PHEWAS of rs140926439. Hematopoietic phenotype was most closely associated.

Figure 1 shows PHEWAS of rs140926439. Hematopoietic phenotype was closely associated. Table 2 shows first ten PHEWAS associations of rs140926439. The strongest association was with acquired hemolytic anemias (p = 0.0021).

## Discussion

Fibronectin is an adhesive molecule that is essential to wound healing, especially to the production of extracellular matrix and reepithelialization. Because of certain functional domains and binding sites in its structure, fibronectin has a wide range of functions during the wound healing process. Fibronectin interacts with the extracellular matrix, cytokines, and other cell types. The creation of extracellular matrix is fibronectin’s primary function. The mature tissue fibronectin that contains extracellular matrix will eventually replace the temporary fibrin-fibronectin matrix that is first formed by plasma fibronectin [6].

The strong association of FN1 SNP rs140926439 with hemolytic anemias suggests a parallel association of rs140926439 with impaired wound healing. Anemic individuals may experience delayed wound closure due to insufficient oxygen supply to healing tissues. Anemia compromises the immune response, making patients more susceptible to wound infections. Collagen synthesis, critical for wound strength, may be impaired in anemic patients. Anemia affects cell proliferation and tissue regeneration, hindering wound closure [7–9].

A derangement of brain wound healing may cause some cases of AD. Wound healing, a highly complex process, has four stages: hemostasis, inflammation, repair, and remodeling. Hemostasis and the initial phases of inflammation in brain tissue are typical of all vascularized tissue, such as skin. However, distinct differences arise in brain tissue during the later stages of inflammation, repair, and remodeling, and closely parallel the changes of AD. Some cases of AD may be due to the initiation of the brain wound healing process, often in the absence of any actual wound. NSAIDS may reduce risk of AD because they potently inhibit wound healing [10].

In conclusion, FN1 appears to be a key player in AD, and its protective variant could offer insights into potential therapeutic targets. However, further research is needed to fully understand the intricate mechanisms underlying AD and to develop effective treatments.

## Figures and Tables

**Table 1.** Relationship of rs140926439 SNP and APOE isoform to AD. When rs140926439 was absent, 0.10% of APOEε2/3 carriers had AD while 0.40% of APOEε4 carriers or homozygotes had AD. This difference was significant (p < 0.001, 2 tail Fisher exact test). When rs140926439 was present, 0.10% of APOEε2/3 carriers had AD while 0.10% of APOEε4 carriers or homozygotes had AD. This difference was insignificant (p = 1).

			APOEε2/3	APOEε4	Total
rs140926439 absent	No AD	Count	302416	105915	408331
		%within APOEε4	99.90%	99.60%	99.80%
	AD	Count	302	462	764
		%within APOEε4	**0.10%**	**0.40%**	0.20%
	Total	Count	302718	106377	409095
rs140926439 present	No AD	Count	2992	1036	4028
		%within APOEε4	99.90%	99.90%	99.90%
	AD	Count	3	1	4
		%within APOEε4	**0.10%**	**0.10%**	0.10%
	Total	Count	2995	1037	4032

**Table 2.** PHEWAS of rs140926439, first ten associations. The strongest association was with acquired hemolytic anemias.

Category	Phenotype	P-value	Effect Size (se)	Number of samples
hematopoietic	Acquired hemolytic anemias	2.10E-03	3.1 (1.0)	135 / 388395
neoplasms	Benign neoplasm of bone and articular cartilage	2.30E-03	1.8 (0.59)	317 / 369610
digestive	Gastrointestinal complications	2.40E-03	1.4 (0.47)	485 / 385685
neoplasms	Colon cancer	3.80E-03	0.51 (0.17)	3108 / 380932
dermatologic	Symptoms affecting skin	4.40E-03	1.2 (0.44)	547 / 401682
musculoskeletal	Kyphoscoliosis and scoliosis	6.30E-03	0.82 (0.30)	1058 / 393240
musculoskeletal	Curvature of spine	6.50E-03	0.79 (0.29)	1125 / 393240
endocrine/metabolic	Mixed hyperlipidemia	7.10E-03	2.1 (0.78)	164 / 371432
respiratory	Bronchitis	7.10E-03	1.1 (0.39)	625 / 373884
mental disorders	Speech and language disorder	7.50E-03	3.3 (1.2)	88 / 406624

## Data Availability

Data available from UK Biobank after approved application
